# Mitochondrial Oxidative Metabolism: An Emerging Therapeutic Target to Improve CKD Outcomes

**DOI:** 10.3390/biomedicines11061573

**Published:** 2023-05-29

**Authors:** Kranti A. Mapuskar, Gabriela Vasquez-Martinez, Gabriel Mayoral-Andrade, Ann Tomanek-Chalkley, Diana Zepeda-Orozco, Bryan G. Allen

**Affiliations:** 1 Free Radical and Radiation Biology Program, Department of Radiation Oncology, University of Iowa Hospitals and Clinics, Iowa City, IA 52242, USA; krantiashok-mapuskar@uiowa.edu (K.A.M.);; 2 Kidney and Urinary Tract Center, The Abigail Wexner Research Institute at Nationwide Children’s Hospital, Columbus, OH 43205, USA; 3 Department of Pediatrics, The Ohio State University, College of Medicine, Columbus, OH 43210, USA

**Keywords:** Chronic Kidney Disease, acute kidney injury, mitochondria, metabolism, reactive oxygen species, superoxide, renal injury

## Abstract

Chronic kidney disease (CKD) predisposes one toward end-stage renal disease (ESRD) and its associated morbidity and mortality. Significant metabolic perturbations in conjunction with alterations in redox status during CKD may induce increased production of reactive oxygen species (ROS), including superoxide (O_2_^●−^) and hydrogen peroxide (H_2_O_2_). Increased O_2_^●−^ and H_2_O_2_ may contribute to the overall progression of renal injury as well as catalyze the onset of comorbidities. In this review, we discuss the role of mitochondrial oxidative metabolism in the pathology of CKD and the recent developments in treating CKD progression specifically targeted to the mitochondria. Recently published results from a Phase 2b clinical trial by our group as well as recently released data from a ROMAN: Phase 3 trial (NCT03689712) suggest avasopasem manganese (AVA) may protect kidneys from cisplatin-induced CKD. Several antioxidants are under investigation to protect normal tissues from cancer-therapy-associated injury. Although many of these antioxidants demonstrate efficacy in pre-clinical models, clinically relevant novel compounds that reduce the severity of AKI and delay the progression to CKD are needed to reduce the burden of kidney disease. In this review, we focus on the various metabolic pathways in the kidney, discuss the role of mitochondrial metabolism in kidney disease, and the general involvement of mitochondrial oxidative metabolism in CKD progression. Furthermore, we present up-to-date literature on utilizing targets of mitochondrial metabolism to delay the pathology of CKD in pre-clinical and clinical models. Finally, we discuss the current clinical trials that target the mitochondria that could potentially be instrumental in advancing the clinical exploration and prevention of CKD.

## 1. Introduction

Mitochondria are crucial for their role as redox sensors and act by interrupting oxidative stress signals, thereby triggering compensatory as well as adaptive responses in target cells. In addition to their role in oxidative phosphorylation and redox regulation, mitochondria mediate several other processes, including inflammation and apoptosis [1]. Since the kidney has one of the highest resting metabolic rates, high mitochondrial mass, and oxygen consumption, mitochondrial health is central to normal kidney metabolism and function [2,3]. Although the kidneys are well-known to be mitochondria-dense, renal mitochondria are known to be heterogenous with different areas in the kidney differing in morphology and density. Studies have shown that the proximal tubule cells that are involved in the reabsorption of nutrients contain larger and more mitochondria relative to distal tubule cells [3]. In addition, medullary mitochondria have a higher oxygen affinity than cortex mitochondria making them suitable to function in low-oxygen environments [4]. In contrast, mitochondria in the renal cortex are relatively dense with an abundant oxygen supply [5]. Alterations in mitochondrial metabolism can result from increased levels of reactive oxygen species (ROS) generating cellular oxidative stress. Under normal physiological conditions, mitochondrial ROS is tightly regulated by the antioxidant and the thiol systems. Since mitochondria are the site of oxidative phosphorylation that generates ATP (adenosine triphosphate), dysfunction in its metabolism can cause alterations in cellular bioenergetics as a result of low ATP production which can contribute to renal pathologies including CKD and diabetic kidney disease [6,7,8]. The pathology of CKD comprises a progressive decline in overall renal function, insulin resistance, increased inflammation, and fibrosis. In addition, increased levels of ROS observed during CKD progression could be a result of hyperactivated NADPH oxidases (NOXs) and increased production of oxidative stress biomarkers including F2-isoprostanes and malonaldehyde [9].

The relationship between mitochondrial perturbations and CKD progression has been studied extensively with the aim to identify and define specific biomarkers that can be followed longitudinally and be therapeutically targeted to delay the onset of CKD. Since mitochondrial dysfunction, depending on its nature, degree, and presentation, may affect the extent of CKD pathology, the interplay between the two often remains abstract. Nevertheless, several hallmark features that define mitochondrial alterations, including mitochondrial oxidative metabolism, biogenesis, and remodeling, are a focus of CKD development. One of the concepts linking CKD and mitochondrial alterations is the unifying hypothesis that focuses on the mitochondria and the pathobiology of diabetic complications [10]. The unifying hypothesis suggests that an increase in the generation of ROS and thus the resulting mitochondrial dysfunction mediate the pathogenesis of microvascular complications in diabetes and the progression to CKD [7,10]. Another aspect linking impaired mitochondrial function and CKD is the association of advanced CKD with muscle atrophy, decreased exercise capacity, and skeletal muscle weakness. These studies suggest that mitochondrial respiratory function is compromised along with reduced muscle mitochondrial mass and decreased energy production in skeletal muscle, which plays a crucial role in the acquired mitochondrial myopathy in CKD [11]. Among other theories linking mitochondria functional decline and CKD progression are depletion in ATP, inflammatory processes, apoptosis, etc. [1,3,12].

This review will discuss mitochondrial metabolism regarding CKD progression and risk factors that could affect CKD progression. Furthermore, this review will also provide insight into mitochondrially targeted therapeutic interventions that are currently clinically available and may improve CKD management. 

### 1.1. CKD and Risk Factors

The prevalence of CKD is around 10–13% worldwide [13,14]. The increasing burden of CKD in the medical community is reflected by the higher number of hospitalizations, increased risk of dialysis, cardiovascular complications, associated financial costs, and mortality [13,14,15,16,17]. The risk of developing ESRD increases with CKD progression. Recent reports have indicated an increase in the prevalence of ESRD from 6–135% from 2006–2012, thus warranting the need for identifying biomarkers during CKD development and progression [18,19]. 

Traditionally, the risk factors for CKD include adverse outcomes of kidney failure, diabetes, hypertension, age, and gender which are causally related to cardiovascular complications [20]. CKD can also significantly add to the risk of cardiovascular events. Studies by Sunström et al. found the rate of cardiorenal events, including CKD and heart failure, were consistently higher than the rates of myocardial infarction or stroke worldwide, in CKD patients [16,21]. Furthermore, cardiorenal-event-related hospital costs were significantly higher than those for atherosclerotic events such as myocardial infarction or stroke worldwide [16]. These results suggest that CKD-related hospitalizations are a leading cost in most countries, followed by cardiac-failure-related events [16]. Increased risk of mortality in CKD patients is commonly associated with kidney-related events, cardiovascular events, acute kidney injury, cognitive decline, and anemia compared to those with normal renal function [22,23]. Recent studies have also reported “non-traditional” or additional risk factors for CKD-based cardiac events including inflammation [24], thrombosis [25], vascular calcification [26], endothelial dysfunction, etc. [20]. 

Diabetes mellitus is a major risk factor for the development of CKD and ESRD. The NIDDK report estimates a total of 34.2 million people (10.5% of the U.S. population) have diabetes with an estimated 26.9 million (8.2%) diagnosed cases [27]. The number of diabetes mellitus cases is expected to rise to 592 million by 2035 with diabetic patients having a greater risk of developing ESRD compared to nondiabetic patients [28]. Furthermore, this study also estimates ESRD being 38% higher in women with diabetes compared to men with diabetes indicating that gender may potentially augment CKD progression [28].

Diabetic nephropathy, also known as diabetic kidney disease (DKD), affects 25–40% of the diabetic patient population. DKD is a microvascular complication associated with structural changes that occur in the glomeruli as a result of hyperglycemia [29,30]. The pathology of DKD includes albuminuria, tubulointerstitial fibrosis, and a progressive decline in kidney function [29,31]. DKD is often a result of mitochondrial dysfunction including an increase in mitochondrial oxygen consumption, intrarenal hypoxia, and increased levels of O_2_^•−^ and thus oxidative damage [29,32]. Recent clinical trials have focused on examining novel therapeutic targets to impede the development of DKD (NCT04074668).

Genetic predisposition is also a significant risk factor for the development of CKD with genome-associated studies identifying loci prone to increased susceptibility for several parameters of kidney function including EGFR, cystatin C, and serum creatinine [33]. Mutations in genes associated with kidney function, including uromodulin as well as polymorphisms in the renin–angiotensin systems, have also been linked to alterations in renal function and risk for developing ESRD [33,34]. Likewise, family members of CKD patients have a high risk of developing CKD. Nearly 23% of dialysis patients report having a close relative with ESRD indicating an urgent need to screen high-risk family members to avoid the potential development of CKD [35].

A progressive decline in renal function occurs with aging due to endogenous comorbidities associated with the aging process increasing the susceptibility of elderly patients to developing CKD. The CREDIT study reported CKD odd ratios of 1.45–2.18 for every ten years of age in subjects over 30 years [36]. The same study also reported increased CKD prevalence in women compared to men [36].

Several other risk factors for CKD progression, including obesity, smoking, socioeconomic status, exposure to nephrotoxins, as well as metabolic factors such as urinary oxalate [37] and FGF23 [38], have been identified [39,40]. 

### 1.2. Kidney Metabolism

The kidney plays a vital role in maintaining glucose levels in the body through processes such as glucose consumption, reabsorption, and gluconeogenesis. The kidneys filter approximately 180 g of glucose daily, most of which is reabsorbed into the circulation [41]. The kidneys take up approximately 10% of the body’s glucose consumption [42]. Most glucose uptake in the kidneys occurs in the distal medullary segments of the nephron that contain glycolytic enzymes in high concentrations. Most of the glucose taken up in this region is oxidized to carbon dioxide and converted to lactate by anaerobic glycolysis [43]. Reabsorption of filtered glucose occurs along the proximal tubule via sodium–glucose cotransporters (SGLTs) and facilitated diffusion of glucose-by-glucose transporter 2 (GLUT2) [44,45,46]. SGL2 processes 90% of the filtered glucose. This is a low-affinity/high-capacity transporter located along the S1 and S2 segments of the luminal surface of the epithelial cells lining the proximal tubule. The high-affinity/low-capacity SGLT1 processes the remaining 10% of the glucose reabsorption in the S3 region of the proximal tubule. SLGT glucose transport is an active process that moves against a concentration gradient and is fueled by the sodium electrochemical gradient and ATP. GLUT2 located in the kidney, on the basolateral membrane of the epithelial renal tubules, is a passive transport system that utilizes equilibrium and releases glucose into the blood, reabsorbed by SGLT1 and SGLT2 [42,47,48].

The kidneys can also make glucose from non-carbohydrate sources such as fatty acids via gluconeogenesis which primarily occurs in the proximal tubular portion of the renal cortex. Free fatty acids can be converted to glucose [43] via four irreversible enzyme-catalyzed reactions which primarily occur in the proximal convoluted tubules (PCTs) [49]. 

Glucose metabolism in the kidney is regulated by hormones such as insulin, glucagon, and catecholamines. These hormones act quickly to alter plasma glucose levels if required by the cell. Insulin suppresses glucose release through gluconeogenesis inhibition and stimulation of renal glucose uptake, glucagon stimulates glucose release through glycogenolysis, and catecholamines cause renal glucose release by stimulating renal gluconeogenesis [42,50].

In addition to glucose, the kidneys efficiently use fatty acids as an energy source. Non-esterified fatty acids are the primary energy source in the kidneys [51]. Most FFA uptake in the kidneys occurs through circulation and synthesis on the basolateral side of the proximal tubule cells [52,53]. In these specialized epithelial cells, there is a high concentration of mitochondria. Here, β-oxidation of fatty acids creates cellular energy via ATP production through the citric acid cycle and oxidative phosphorylation. The FFA route into the cell across the cellular membrane is facilitated by CD36 and fatty-acid binding protein but can also cross over through endocytosis of albumin-associated fatty acids [51]. FFAs can also enter the cells of the proximal tubules from the apical side through reabsorption [53]. If there is an excess of FFAs available to the cells, they can be esterified with glycerol and packaged as triglycerides in lipid droplets inside the cell. These stored energy sources can be utilized rapidly when the cell requires fuel [54]. FFAs are carried into the cell on albumin which is then degraded in lysosomes. If energy is needed, the FFAs are channeled to the β-oxidation process of the mitochondria. If energy is not required, the FFAs are stored as triglyceride lipid droplets [53].

## 2. Mitochondrial Function in Normal Renal Metabolism

Due to high metabolic demands, the resting metabolic rate of the kidney is second to the heart and as such requires an abundance of mitochondria to be able to generate ATP to meet its energy demands. Renal mitochondria are known to play a plethora of significant roles in maintaining kidney homeostasis and metabolism including ATP production, regulation of oxidative stress levels, apoptosis, autophagy, calcium homeostasis, etc.

### 2.1. Mitochondria and ATP

Most of the ATP generated in the kidney is from the mitochondrial electron transport chain (ETC), a highly regulated process that occurs on the inner mitochondrial membrane. The ATP thus generated is required for a variety of cellular functions including the active transport of nutrients [55]. In general, all renal cells require ATP to maintain cellular homeostasis; however, the ATP generation mechanism in the kidney is dependent on the specific cell type. For example, ATP generated in the proximal tubules in the renal cortex is dependent on ETC for the transport of glucose and nutrients [3,55]. By contrast, podocytes, glomerular cells, mesangial cells, and endothelial cells have a relatively lower oxidative capability [3,56]. Furthermore, proximal tubule cells produce ATP primarily by aerobic respiration due to their high energy demand [57].

### 2.2. Mitochondrial ETC and ROS

ATP generated in the mitochondria is via the mitochondrial ETC by a process called oxidative phosphorylation (OXPHOS) [58,59]. An increase in the residence time of electrons in specific sites of the mitochondrial ETC can lead to one-electron reductions of oxygen to superoxide (a form of ROS); most are converted to H_2_O_2_ by superoxide dismutases to form H_2_O and O_2_ [60]. Thus, mitochondria play a pivotal role in the regulation of oxidative stress by maintaining the cellular redox balance.

### 2.3. Mitochondrial Apoptosis

Mitochondrial apoptotic signaling is a tightly regulated process and disruptions in the regulation of the apoptotic process can contribute to a variety of pathologies including CKD development and progression in the kidney; mitochondrial dysfunction can contribute to apoptotic cell death and the progression of kidney disease. The permeabilization of the outer membrane of the mitochondria (MMP) regulated by the Bcl-2 family is responsible for the release of pro-apoptotic factors from the intermembrane space into the cytoplasm [61]. As a result of MMP, cytochrome C is released from the mitochondria into the cytoplasm where it interacts with apoptotic protease-activating factor 1 (Apaf-1) and activates the initiator caspase, caspase-9, in the apoptosis pathway [61]. Furthermore, mitochondria also harbor the mitochondrial permeability transition pore (MPTP), which is a channel that can be induced by calcium overload, proapoptotic signals, and oxidative stress [62,63]. An imbalance of the MPTP has been shown to cause mitochondrial swelling, disruption of the membrane potential, and cell death [64,65]. 

Apart from ATP production, maintenance of redox balance, and regulation of apoptosis, renal mitochondria are also involved in calcium homeostasis and autophagy/mitophagy. Overall, the maintenance of healthy mitochondria is pivotal for redox homeostasis, and cellular health in the kidney. Alterations in mitochondrial metabolism and function can significantly contribute to kidney dysfunction and the development and/or progression of CKD.

### 2.4. Mitochondrial Components in Chronic Kidney Disease (CKD)

Mitochondria originated from the incorporation of a prokaryotic organism into a eukaryote more than 1500 million years ago [66]. Mitochondria contain approximately 1500 proteins of which 13 are encoded by the nuclear genome [67]. Interestingly, 99% of mitochondrial proteins are synthesized by cytosolic ribosomes [67]. The mitochondria double-stranded genome encodes for 13 proteins involved in oxidative phosphorylation. [68]. 

Mitochondria play a bioenergetic role in eukaryotic cells with the synthesis of adenosine triphosphate by oxidative phosphorylation, lipid, and amino acid biosynthesis; generate reactive oxygen species (ROS) [69]; and participate in calcium homeostasis [70], heme biosynthesis, fatty acid oxidation, activation of the stress response of the endoplasmic reticulum, and apoptosis [71,72]. The metabolism of carbohydrates, fats, and proteins is connected to the Krebs cycle, where the acetyl CoA is generated. Acetyl CoA is oxidized and produces electron donor molecules, such as NADH and FADH, which are transferred to the mitochondrial respiratory chain to initiate the process of oxidative phosphorylation and ATP synthesis through protein complexes embedded in the mitochondrial inner membrane. 

**Figure 1:**
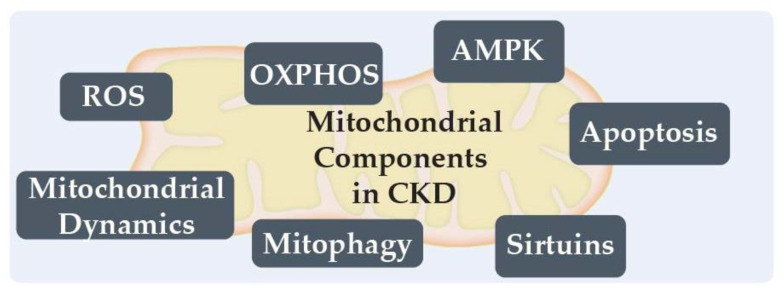
Mitochondrial Components in CKD.

Mitochondrial dysfunction is a common feature of CKD, represented by increased oxidative stress levels, changes in the mitochondrial number and morphology, increased sensitivity of apoptosis, instability to the mitochondrial DNA, alterations in the renal microvasculature, inflammation, and fibrosis (Figure 1). The energy state of cells is associated with specific changes in mitochondrial morphology. These dynamic changes are fusion, fission, transport, and mitophagy [73]. For example, mitochondrial fusion is a two-step process that involves the fusion of the inner and outer membranes and is mediated by three GTPases: dynamin mitofusin 1 (MFN 1), MFN 2, and optic atrophy 1 (OPA1) [74]. Mitofusins are integral proteins that participate in the fusion of the outer membrane, while Opa1 mediates the fusion of the inner membrane [74,75]. Fusion improves oxidative phosphorylation and decreases the number of damaged mitochondria by combining their contents with healthy mitochondria during mitochondrial injury [75]. 

Mitochondrial fission or fragmentation has been associated with impaired oxidative phosphorylation and regeneration of ROS (Figure 1). The primary function of fission is to isolate damaged mitochondria from the mitochondrial network for further degradation through mitophagy [76]. Several studies have linked an increase in fission after AKI and the progression of diabetic kidney disease (DKD) [77,78]. Dynamin-related protein 1 (DRP1) inhibition decreases tubular injury and the progression of kidney injury [79]. Finally, mitophagy is a selective form of autophagy that specifically eliminates damaged or dysfunctional mitochondria. Mitophagy is induced under conditions of cellular stress and contributes to the adaptive mechanism for maintaining a population of functional mitochondria.

After the initial injury, the surviving tubular epithelial cells undergo dedifferentiation for proliferation and renal repair. In this process, if the damage is not as severe and allows good repair, the integrity and function of the tubular epithelium can be restored. However, incomplete or maladaptive repair results in the development of chronic pathologies such as interstitial fibrosis or progression to CKD [80]. The arrest of the cell cycle G2/M alters mitochondrial biogenesis (MB) and promotes the alteration in mitochondrial DNA. Similarly, senescence alters cell apoptosis and stimulates tubular epithelial cells to a secretory phenotype of cytokines, chemokines, and growth factors that favor inflammation and fibrosis [81].

Changes during a mitochondrial injury in the process of MB have been studied by regulatory molecules such as peroxisome proliferator-activated receptor-γ coactivator-1 α (PGC-1 α), AMP-activated protein kinase (AMPK), and nuclear respiratory factors 1 and 2 (NRF1 and NRF2) [82]. It has been observed that when mitochondrial damage is severe, instead of MB increasing its metabolism and replacing injured or dysfunctional mitochondria, MB is altered and participates in the maladaptive process resulting in fibrosis [83]. Loss in tubular cells of PGC-1α and mitochondrial transcription factor A (TFAM), which are transcriptional regulators of MB, increased susceptibility to AKI and renal fibrosis.

Changes in ATP production are affected after kidney injury as they increase free radicals (ROS). Although ROS are also essential for cell function [84], high concentrations are toxic to mitochondria, generating alterations of DNA, proteins, and lipids. These variations can cause a decrease in electron transfer, reduction of intracellular levels of ATP, and modifications in the mitochondrial membrane pore with loss of membrane potential originating, thus, cell death [63]. Furthermore, the oxidation of fatty acids stops temporarily during kidney injury [85]. ATP synthesis could be due to the oxidation of fatty acids since the nephrons have a high mitochondria content [86]. If the accumulation of lipids persists, it can affect the mesenchymal reprogramming of renal epithelial cells, ultimately increasing the risk of developing CKD.

In addition to the direct effects of ROS on the progression of CKD, several other pathways regulate the pathophysiology of kidney disease. Several studies have shown that the SIRT family of nicotinamide-adenine-dinucleotide-dependent histone deacetylases (SIRT1-7) plays a crucial role in renal protection via the alleviation of oxidative stress levels, fibrosis, and inflammation [87,88,89]. SIRT1, a highly conservative nuclear/cytoplasmic protein expressed in kidneys, facilitates the acetylation and regulation of the activity of several transcription factors by nuclear-cytoplasmic shuttling [90]. As a negative regulator of p53, SIRT1 can induce apoptosis and cellular senescence following oxidative stress [91]. SIRT1 has also been shown to play a role in renal fibrosis via the downregulation of matrix metalloproteinase-14 (MMP14) [92]. Furthermore, SIRT1 also regulates kidney fibrosis by Smad4 deacetylation and the inhibition of matrix metalloproteinase-7 expression in tubular epithelial cells, thus attenuating renal fibrotic processes [93]. In addition to SIRT1, SIRT3, which is a mitochondrial deacetylase [94], has been shown to be crucial in regulating the stress-associated renal response. Kidneys of Sirt3^−/−^ mice demonstrated increased levels of oxidative stress, enhanced lipid accumulation, and structural damage to the tubular cells as well as a decline in mitochondria mass and ATP production compared to WT mice, suggesting another mechanistic link between mitochondrial function and development of kidney disease [95].

AMP-activated protein kinase (AMPK) is a ubiquitous cellular energy sensor that serves as a crosslink between energy metabolism, growth, inflammation, and stress [96,97]. It has been reported to decrease in several organs including the kidney following a high-fat diet and could potentially contribute to the initial events of CKD development [96,97]. Furthermore, the structural and functional changes that contribute to the pathology of CKD can result in chronic energy deprivation and metabolic stress which in turn can activate AMPK [98]. Inhibition of renal AMPK has also been associated with poor prognosis indicated by inflammation, insulin resistance, fibrosis, and CKD [98,99]. AMPK could thus serve as a therapeutic target that could be potentially exploited to delay the development and progression of CKD.

Nuclear factor erythroid 2-related factor 2 (NRF2) regulates oxidative damage by inducing the expression of its target genes by binding the antioxidant response element (ARE) [100]. Endogenous NRF2 activation, as well as repression, has been observed in CKD patients depending upon the stage, severity, and comorbidities of CKD. Robust activation of NRF2 is observed in the earlier stages of CKD development along with inflammation. In contrast, advanced CKD has been associated with the repression of the NRF2 pathway [101]. A patient-based study conducted by Rasmussen et al. reported a biphasic pattern of NRF2 concentration in CKD wherein low glomerular filtration rates were associated with lower NRF2 protein concentration [101]. The study reported increased NRF2 protein levels in mild to moderate kidney function impairment, and the downregulation of NRF2 was observed in severe renal impairment [101].

## 3. Pathology of AKI to CKD Transition

The pathophysiological mechanisms of the transition from acute kidney injury (AKI) to CKD include vasoconstriction, congestion of microvasculature [102], inflammation and infiltration of monocytes, macrophages, neutrophils, T- and B-cell activation [103], dysregulated tissue repair, and, finally, fibrosis with increased proliferation of myofibroblasts [104]. The transition from AKI to CKD is being widely studied. The transition from acute kidney injury to CKD is being widely studied. It can be summarized in four primary events: tubular cell cycle arrest, endothelial response, inflammatory response, and, finally, fibrosis.

### 3.1. Tubular Cell Cycle Arrest

Tubular cell cycle arrest occurs during AKI and has a crucial role in the protection of renal tubular epithelial cells as well as in maladaptive repair following AKI [105]. Although the cell cycle arrest at the G1 phase of the cell cycle halts damaged DNA replication, sustained cell cycle arrest can present in severe AKI which can ultimately result in cellular senescence and maladaptive repair [105]. Tissue inhibitor metalloproteinase 2 (TIMP-2) and insulin-like growth factor binding protein 7 (IGFBP7), markers of cell cycle arrest, are predicted to represent “stress” prior to the development of AKI injury [106,107]. These biomarkers subsequently can trigger an autocrine or paracrine response and lead to the release of profibrotic factors and ultimately contribute to the development of fibrosis [108]. Thus, regulating this early phase of cell cycle impairment can thus prove to be a vital therapeutic target for delaying/preventing the transition of AKI to CKD. 

### 3.2. Endothelial Response

After a renal insult, endothelial alterations are one of the first responses starting with a capillary rarefaction that produces more regional hypoxia, ischemia, inflammation, and necrosis [109]. The decrease of angiogenic factors such as endothelial growth factor (VEGF), a cytokine crucial for preserving vascular networks, causes microvascular dysfunction and morphological changes in the nephron [110]. In some animal models with induced renal injury and inactivation of sirtuin 1 (Sirt-1) in endothelial cells, increased kidney damage, injury progression, and fibrosis were observed [111]. Transforming growth factor-beta (TGF-β), a cytokine involved in cellular processes such as hematopoiesis, proliferation, angiogenesis, cell differentiation, migration, and apoptosis, contributes to the endothelial-to-mesenchymal transition, which favors fibrosis and chronic damage.

#### 3.2.1. Inflammatory Response

After the inflammation during AKI, there is an infiltration of circulating immune cells (T and B cells) which are attracted by the damage-associated molecular patterns (DAMPs) and cytokines of the injured renal cells. This profibrotic environment activates pericytes to proliferate and evolve into myofibroblasts, developing fibrosis and chronic kidney disease [112].

#### 3.2.2. Fibrosis

Myofibroblasts can be derived from resident fibroblasts or pericytes. They are responsible for the production of the extracellular matrix, with the collagen fibers deposition, fibronectins, TGF-β, and other glycoproteins contributing to fibrosis [113]. The expression of α-smooth muscle actin (α-SMA), usually compacted to the vascular compartment, and platelet-derived growth factor-β receptor (PDGFR-β) identifies these cells in the kidney interstitium, injured [114].

Studies that help us understand the molecular mechanisms in the development and transition from AKI to CKD are paramount since they would aid in identifying new strategies for treating and preventing CKD.

### 3.3. Targeting Mitochondrial Oxidative Metabolism in Chronic Kidney Disease (CKD)

ATP synthesis and oxidative metabolism play an essential role in mitochondrial dysfunction during acute kidney injury (AKI) and the progression to CKD [2,7]. Increased generation of reactive oxygen species (ROS), predominantly by the electron transport chain (ETC) through oxidative phosphorylation, has been correlated with mitochondrial dysfunction, causing cellular damage and resulting in the progression of kidney disease [8]. Mitochondria have thus been considered a therapeutic target in CKD by attempting to alleviate mitochondrial function and thus diminish cellular damage.

Several studies have explored the mitochondria as a critical target to improve the outcomes of patients with CKD via preserving the mitochondrial structure and inhibiting mitochondrial oxidative damage, thus preventing CKD progression [7,78,80,115,116]. The use of novel mitochondrially targeted drugs has been partially explored in different injury animal models [80], including cisplatin [116,117,118], unilateral ureteral obstruction (UUO) [119], ischemia-reperfusion [120], diabetic nephropathy [121], sepsis [122], hypertension-related vascular injury [123] and before kidney transplantation [124]. However, compounds that can specifically target the mitochondria, capable of crossing the blood–brain barrier, with a known dose reported to be effective in vivo, are still needed (Table 1).

### 3.4. MitoQ

Studies have shown that Coenzyme Q10 (CoQ10), a fat-soluble vitamin-like quinone and antioxidant, preserves mitochondrial function [140] and restores renal function impaired by diabetic nephropathy (DN) [121]. Mitochondrial coenzyme Q (MitoQ), a form of CoQ10, is a ubiquinone derivative, with the ability to target mitochondria, by binding to a lipophilic triphenylphosphonium (TPP) cation to achieve mitochondrial targeting [141,142]. Due to membrane potential, the cation accumulates within the mitochondria, and ubiquinone is reduced to ubiquinol, attenuating lipid peroxidation, and protecting isolated mitochondria from oxidative damage [125] (Table 1). In the context of CKD, MitoQ has been shown to prevent renal dysfunction and reduce urinary albumin, interstitial fibrosis, and glomerular damage by decreasing ROS [137,143]. MitoQ can also attenuate inflammation by reducing leukotriene (LT) B4 and myeloperoxidase (MPO) [144]. Studies have reported that the synergism between MitoQ and CoQ10 supplements can mildly suppress mitochondrial ROS levels, suggesting that MitoQ alone could be more effective than CoQ10 [145,146]. The renin–angiotensin system (RAS) activation is commonly present in CKD. The angiotensin II-induced podocyte injury model is known to cause proteinuria and initiates the progression of CKD. Zhu et al. demonstrated that MitoQ could ameliorate oxidative stress, apoptosis, and mitochondrial fission through Nrf2 signaling in Ang II-stimulated podocyte injury both in vivo and in vitro [126].

### 3.5. Mito-TEMPO

Mito-TEMPO is a mitochondrially targeted superoxide mimetic with superoxide and alkyl radical scavenging properties known to alleviate renal fibrosis [127] (Table 1). Furthermore, it has been shown that mito-TEMPO treatment can also lessen podocyte injury and loss in diabetic nephropathy [128]. Studies have also reported that mito-TEMPO can improve mitochondrial function by inhibiting mitochondrial ROS (mtROS), restoring the renal cell’s mitochondrial DNA (mtDNA), reducing the accumulation of renal interstitial collagen and renal fibrosis, and increasing the generation of ATP, suggesting mito-TEMPO reverses mitochondrial impairment by superoxide reduction [127,129]. Finally, mito-TEMPO has been reported to attenuate mitochondrial dysfunction, inhibit inflammatory cytokine expression, and suppress endoplasmic reticulum stress in a nephrectomy-induced renal fibrosis mouse model [127,147,148].

### 3.6. SS-31

Szeto–Schiller (SS) peptides are molecules constituted by aromatic residues and basic amino acids. Their uptake is not dependent on mitochondrial membrane potential, and they are known as potent antioxidants due to tyrosine or 2,6-dimethyl-L-tyrosine (Dmt) residues [120]. The mitochondrial-inner-membrane-targeted SS-31 (d-Arg-2′, 6′-dimethyltyrosine-Lys-Phe-NH2) improved renal function by increasing recovering ATP, scavenging ROS (superoxide, hydrogen peroxide, peroxynitrite, and hydroxyl radicals) [149], and suppressing mitochondrial permeability in CKD model with a right nephrectomy following the treatment of hypochlorite-modified albumin in a remnant-kidney rat model [150,151]. It was previously reported that SS-31 protects against high-glucose-induced renal injury in diabetic nephropathy, inhibiting transforming-growth factor (TGF)-β1, NOX4, and thioredoxin-interacting protein (TXNIP) [130] (Table 1). Furthermore, Szeto, HH et al. reported that SS-31 treatment after ischemia preserves the mitochondrial structure, facilitates electron transport, reduces ROS production, and pauses the progression of glomerulosclerosis and tubulointerstitial fibrosis [131].

In addition, SS-31 has been shown to interact with cardiolipin (an anionic phospholipid on the inner mitochondrial membrane required for cristae formation) to help maintain the structural integrity of the mitochondria and function. A study carried out by Birk et al. in 2013 showed that SS-31 binds to and stabilizes cardiolipin, preventing cytochrome c peroxidase activity that catalyzes cardiolipin peroxidation and results in mitochondrial damage during ischemia [152]. They also demonstrated that pretreatment with SS-31 protects cristae membranes and prevents mitochondrial swelling [152].

In other studies, SS-31 has been shown to improve mitochondrial coupling and ATP production in the muscles of aged mice [153]. Furthermore, treatment with SS-31 also restored redox homeostasis by reducing the levels of oxidant production as measured by 4-hydroxynonenal and thiol levels, alleviated energy deficits, and improved skeletal muscle function [153,154].

### 3.7. MitoSOD

In 2012, Kelso, G.F. et al. developed a mitochondria-targeted (SOD) mimetic, MitoSOD, by conjugating the core functionality of the Mn(II) macrocycle SOD mimetic M40403 to the mitochondria-targeting lipophilic cation TPP. MitoSOD accumulates into mitochondria and scavenges mitochondrial O_2_^•−^, thereby protecting against oxidative damage [132,133] (Table 1). MitoSOD has been used in gentamicin-mediated nephropathy in rats and showed that M40403 prevents nitrotyrosine formation, poly (ADP-ribose) synthetase activation, and tubular necrosis; however, the use of MitoSOD has not been reported in in vivo models [134].

### 3.8. Avasopasem Manganese

Avasopasem manganese (AVA a.k.a. GC4419) is a mitochondrially targeted SOD mimetic (66 that recently completed a Phase 3 trial investigating the Effects of GC4419 on Radiation-Induced Oral Mucositis in Head/Neck Cancer Patients (NCT03689712). The overall goal of the trial was to compare the efficacy and safety of GC4419 with a placebo to reduce the duration, incidence, and severity of severe OM (SOM). Preclinically, our group was the first to report the effect of this superoxide-specific SOD mimetic in cisplatin-induced kidney injury and its role in CKD progression in murine models of renal injury [116]. We demonstrated that GC4419 attenuated cisplatin-induced CKD, ameliorating oxidative damage and renal fibrosis one month after injury. More interestingly, retrospective analysis of renal function and biomarkers in a subset of head and neck cancer patients receiving high-dose cisplatin and radiation in a double-blinded, placebo-controlled clinical trial (NCT02508389) demonstrated that a 90 mg AVA treatment prevented a significant reduction in estimated glomerular filtration rate (eGFR) three months as well as six and twelve months after treatment compared to 30 mg AVA and placebo [118] (NCT03689712) (Table 1). Furthermore, the study also shows that treatment with 90 mg of AVA could potentially contribute to renal repair following cisplatin treatment as seen by an increase in epithelial growth factor (EGF), which is known to aid in renal recovery [118,155], supporting the hypothesis that a selective dismutase mimetic could offer the potential for kidney protection following a high-dose cisplatin treatment.

### 3.9. SRT1720

The activators of mitochondria biogenesis can be promising therapeutic targets. SRT1720 (N-[2-[3-(piperazin-1-ylmethyl) imidazo [2,1-b][1,3]thiazol-6-yl]phenyl]quinoxaline-2-carboxamide), an SIRT1 activator, has an essential role in preventing the progression of CKD by transforming growth factor-beta1 (TGF-beta1)/connective tissue growth factor (CTGF) pathway inhibition in UUO-induced tubulointerstitial fibrosis [135] (Table 1). Studies performed by Han et al. in renal proximal tubular epithelial (HK-2) cells treated with high glucose in the presence of 2.5 µM of SRT1720 showed the restoration of SIRT1 expression and activity [136] (Table 1). Furthermore, the study also demonstrated that SRT1720 improved renal function, attenuated glomerular hypertrophy, and interstitial fibrosis, as well as inhibited TGFβ1 and nuclear factor κB (NF-κB) activation in diabetic mice [136]. Additionally, SRT1720 also suppressed HIF1α, GLUT1, and SNAIL expressions both in vivo and in vitro, indicating a role for SRT1720 in the prevention of diabetes-induced renal fibrosis via the SIRT1/HIF1α/GLUT1/SNAIL pathway [136].

### 3.10. Sulforaphane (SFN)

Sulforaphane (SFN) modulates mitochondrial function and dynamics [156]. SFN activates cellular antioxidants such as superoxide dismutase (SOD), catalase, and the reduced forms of glutathione (GSH) and glutathione reductase. Studies have reported that it protects the kidneys from UUO-induced renal oxidative stress by PI3K/Akt/GSK3β activity. The effect of SFN has been attributed to significantly increased nuclear Nrf2 translocation and decreased mitochondrial Bax translocation and cytochrome C release [137] (NCT04608903) (Table 1). In the recent past, SFN has been administered as broccoli sprout extract (BSE) in an interventional clinical trial (NCT04858854) in patients with CKD with type 2 diabetes. There are ongoing phase 1 and 2 clinical trials at the University of Rochester Medical Center, Rochester, New York that focus on testing the safety of the sulforaphane in CKD (NCT05153174).

Several other small-molecule pharmacological compounds have been utilized to target mitochondrial metabolism in the context of CKD and renal injury in general including celastrol (CLT) a.k.a tripterygium wilfordii, a traditional Chinese medicinal plant that exerts anti-inflammatory effects by inhibiting pro-inflammatory cytokines and chemokines and reduces endothelial damage to alleviate the severity of CKD [138] (Table 1). Studies have also shown CLT to increase the levels of nitric oxide (NO) by increasing endothelial nitric oxide synthase (eNOS) whilst inhibiting VCAM-1, thus decreasing inflammation in a murine model of CKD [138]. Curcumin, another traditional herb and dietary spice, is also known to have anti-inflammatory, antioxidant, and anti-fibrotic properties that can help ameliorate CKD [139]. Curcumin has been shown to reduce the levels of inflammatory molecules, inhibit ROS levels as well as inhibit the activation of fibrotic pathways, reduce the accumulation of extracellular matrix components, and attenuate kidney fibrosis [139]. Curcumin also demonstrates renal protection by reducing proteinuria and alleviates structural abnormalities associated with CKD progression [139] (Table 1).

Understanding the various pathways that govern the mitochondrial function and deciphering its role in kidney diseases in conjunction with mitochondrially targeting agents could identify a therapeutic window that could be targeted to significantly impact the treatment of renal diseases and CKD progression.

## 4. Conclusions

In the recent past, unified preclinical and clinical studies have facilitated the understanding of CKD risks, development, and pathogenesis, thereby revealing novel therapeutic targets. The attribution of increased ROS levels to CKD development, although debatable, is a common theme of mitochondrial dysfunction, and alterations in mitochondrial oxidative metabolism do emerge from past studies. Assessing specific biomarkers of renal function during CKD development and progression using pharmacological interventions could help prevent and delay CKD pathology. Several therapeutic interventions that are mitochondrially targeted are currently in clinical trials to prevent, treat, as well as delay CKD progression.

## Figures and Tables

**Table 1:** Summary of mitochondrial oxidative metabolism targets in Chronic Kidney Disease, their mechanism of action and related clinical trials.

Compound	Mechanism	Clinical Trial
Mitochondrial coenzyme Q (MitoQ)	Targets mitochondria using the lipophilic triphenylphosphonium (TPP) cation, attenuates lipid peroxidation [125], ameliorates oxidative stress, apoptosis, and mitochondrial fission through Nrf2 signaling [126]	
MitoTEMPO	Mitochondrially-targeted piperidine nitroxide with the ability to regulate the coenzyme Q pool within mitochondria [127]. Known to prevent renal fibrosis [127,128,129], alleviates podocyte injury [84], reduces accumulation of interstitial collagen [127,128,129]	
SS-31 (MTP-131, Elamipretide, and Bendavia)	Inner mitochondrial membrane-targeted [88] Szeto-Schiller (SS) peptide that improves renal function by increasing ATP and scavenging ROS [130,131], Inhibits TGFβ1, NOX4, and TXNIP in glucose-induced renal injury [130]	NCT02436447
MitoSOD	Accumulates in mitochondria and scavenges mitochondrial superoxide [132,133], prevents nitrotyrosine formation [134].	
Avasopasem Manganese (GC4419)	Selectively catalyzes the dismutation of O_2_^•−^ to hydrogen peroxide (H_2_O_2_) [118], protects against Cisplatin-Induced AKI and AKD [116,117,118], protects against cisplatin-induced CKD [67]	NCT05412472 NCT04529850 NCT03689712
SRT1720	Selective, synthetic Sirt1 activator that promotes the binding of NAD+ to the catalytic site of the enzyme [135,136]	
Sulforaphane (SFN)	A natural compound found in cruciferous vegetable. Inhibits the KEAP-1-mediated degradation of Nrf2, allowing binding to the antioxidant response element (ARE) and inducing transcription of target genes thereby reducing ROS, inflammation and fibrosis [137]	
Celastrol (CLT)	Inhibits pro-inflammatory cytokines and chemokines and reduces endothelial damage [138]	NCT04981613 NCT00518362
Curcumin	Anti-inflammatory, antioxidant properties. Blunts NOX and Xanthine Oxidase expression leading to a reduction in oxidative stress [139]	NCT03475017 NCT04413266 NCT03223883 NCT01831193

## Data Availability

Not applicable.

## Abbreviations

Superoxide Dismutase (SOD), Avasopasem Manganese (AVA or GC4419), Estimated Glomerular Filtration Rate (eGFR), Acute Kidney Injury (AKI), Chronic Kidney Disease (CKD), End Stage Renal Disease (ESRD), Reactive Oxygen Species (ROS), Electron Transport Chain (ETC), Superoxide (O_2_^•−^), Hydrogen Peroxide (H_2_O_2_), Neutrophil Gelatinase-Associated Lipocalin (NGAL), Kidney Injury Molecule-1 (KIM-1), Epithelial Growth Factor (EGF), sodium-glucose cotransporters (SGLTs), glucose transporter2 (GLUT2), NADPH Oxidases (NOXs), proximal convoluted tubule (PCT), Diabetic Kidney Disease (DKD), Mitofusins (MFNs), Optic Atrophy 1 (OPA1), Dynamin-related protein 1 (DRP1), Mitochondrial coenzyme Q (MitoQ), Adenosine triphosphate (ATP), triphenylphosphonium (TPP), Transforming Growth Factor-beta1 (TGF-beta1), Connective Tissue Growth Factor (CTGF), Sulforaphane (SFN), Diabetic Nephropathy (DN), Sirtuin 1 (SIRT1).
